# EpiDiP/NanoDiP: a versatile unsupervised machine learning edge computing platform for epigenomic tumour diagnostics

**DOI:** 10.1186/s40478-024-01759-2

**Published:** 2024-04-04

**Authors:** Jürgen Hench, Claus Hultschig, Jon Brugger, Luigi Mariani, Raphael Guzman, Jehuda Soleman, Severina Leu, Miles Benton, Irenäus Maria Stec, Ivana Bratic Hench, Per Hoffmann, Patrick Harter, Katharina J Weber, Anne Albers, Christian Thomas, Martin Hasselblatt, Ulrich Schüller, Lisa Restelli, David Capper, Ekkehard Hewer, Joachim Diebold, Danijela Kolenc, Ulf C. Schneider, Elisabeth Rushing, Rosa della Monica, Lorenzo Chiariotti, Martin Sill, Daniel Schrimpf, Andreas von Deimling, Felix Sahm, Christian Kölsche, Markus Tolnay, Stephan Frank

**Affiliations:** 1 Institut für Medizinische Genetik und Pathologie, Universitätsspital Basel, Schönbeinstr. 40, 4031 Basel, Switzerland; 2 Klinik für Neurochirurgie, Universitätsspital Basel, Spitalstrasse 21, 4031 Basel, Switzerland; 3 Human Genomics, Institute of Environmental Science and Research (ESR), 5022 Porirua, Wellington, New Zealand; 4 Life&Brain GmbH, Venusberg-Campus 1, Gebäude 76, 53127 Bonn, Germany; 5 Institute of Neuropathology, Center for Neuropathology and Prion Research, Feodor- Lynen-Str. 23, 81377 München, Germany; 6 Neurological Institute (Edinger Institute), University Hospital, Heinrich-Hoffmann- Straße 7, 60528 Frankfurt am Main, Germany; 7 German Cancer Consortium (DKTK), Partner Site Frankfurt, German Cancer Research Center (DKFZ), Heidelberg, Germany; 8 Frankfurt Cancer Institute (FCI), Frankfurt am Main, Germany; 9 Institute of Neuropathology, University Hospital Münster, Pottkamp 2, 48149 Münster, Germany; 10 Forschungsinstitut Kinderkrebszentrum, Martinistrasse 52, 20251 Hamburg, Germany; 11 Department of Pediatric Hematology and Oncology, University Hospital Hamburg- Eppendorf, Hamburg, Germany; 12 Institute of Neuropathology, University Hospital Hamburg-Eppendorf, Hamburg, Germany; 13 Department of Neuropathology, Department of Neuropathology, Charité– Universitätsmedizin Berlin, Charitéplatz 1, 10117 Berlin, Germany; 14 Institut universitaire de pathologie, Lausanne University Hospital (CHUV), University of Lausanne, Rue du Bugnon 25, 1011 Lausanne, Switzerland; 15 15. Luzerner Kantonsspital, Pathologie, Haus 27, 6000 Spitalstrasse, Luzern 16, Switzerland; 16 Klinik für Neurochirurgie, Luzerner Kantonsspital, Haus 31, 6000, 16 Spitalstrasse, Luzern, Switzerland; 17 Medica Pathologie Zentrum Zürich, Hottingerstrasse 9 / 11, 8032 Zürich, Switzerland; 18 CEINGE-Biotecnologie Avanzate, Via Gaetano Salvatore, 486 - 80145 Napoli, Italy; 19 Hopp Children’s Cancer Center Heidelberg (KiTZ), Heidelberg, Germany; 20 Division of Pediatric Neurooncology, German Consortium for Translational Cancer Research (DKTK), Im Neuenheimer Feld 280, 69120 Heidelberg, Germany; 21 Department of Neuropathology, Institute of Neuropathology, University Hospital Heidelberg, Im Neuenheimer Feld 280, 69120 Heidelberg, Germany; 22 CCU Neuropathology, German Consortium for Translational Cancer Research (DKTK), Im Neuenheimer Feld 280, 69120 Heidelberg, Germany; 23 23. DKFZ, Im Neuenheimer Feld 280, 69120 Heidelberg, Germany; 24 Pathologisches Institut der LMU, Thalkirchner Str. 36, 80337 München, Germany

**Keywords:** Digital pathology, Tumour, Oncology, Methylation, Methylome, Unsupervised machine learning, Artificial intelligence, Same-day classification, Intraoperative, UMAP, Dimension reduction, Nanopore sequencing, Microarray, Methylation sequencing, Epigenetics, Copy number profiling, Edge computing, gpGPU, SoC, Cryptocurrency miner

## Abstract

DNA methylation analysis based on supervised machine learning algorithms with static reference data, allowing diagnostic tumour typing with unprecedented precision, has quickly become a new standard of care. Whereas genome-wide diagnostic methylation profiling is mostly performed on microarrays, an increasing number of institutions additionally employ nanopore sequencing as a faster alternative. In addition, methylation-specific parallel sequencing can generate methylation and genomic copy number data. Given these diverse approaches to methylation profiling, to date, there is no single tool that allows (1) classification and interpretation of microarray, nanopore and parallel sequencing data, (2) direct control of nanopore sequencers, and (3) the integration of microarray-based methylation reference data. Furthermore, no software capable of entirely running in routine diagnostic laboratory environments lacking high-performance computing and network infrastructure exists. To overcome these shortcomings, we present EpiDiP/NanoDiP as an open-source DNA methylation and copy number profiling suite, which has been benchmarked against an established supervised machine learning approach using in-house routine diagnostics data obtained between 2019 and 2021. Running locally on portable, cost- and energy-saving system-on-chip as well as gpGPU-augmented edge computing devices, NanoDiP works in offline mode, ensuring data privacy. It does not require the rigid training data annotation of supervised approaches. Furthermore, NanoDiP is the core of our public, free-of-charge EpiDiP web service which enables comparative methylation data analysis against an extensive reference data collection. We envision this versatile platform as a useful resource not only for neuropathologists and surgical pathologists but also for the tumour epigenetics research community. In daily diagnostic routine, analysis of native, unfixed biopsies by NanoDiP delivers molecular tumour classification in an intraoperative time frame.

**Supplementary Information:**

The online version contains supplementary material available at 10.1186/s40478-024-01759-2.

## Introduction

Epigenetic analyses of tumour tissue have become increasingly important in personalised oncology and have recently been defined by the WHO as a standard-of-care principle in the classification of central nervous system tumours [30]. Genome-wide copy number profiles obtained in parallel to DNA methylation profiles further contribute to molecular tumour diagnostics [2, 6]. Currently, artificial intelligence-driven methylome analysis and copy number profiling can justifiably be considered the most advanced and clinically relevant branch of digital pathology [15]. In this context, we termed our resource web service “epigenomic digital pathology / EpiDiP” and refer to the locally installed software built around 3rd generation sequencing as “nanopore digital pathology / NanoDiP”.

A number of supervised epigenetic classification systems have been proposed and, in part, made publicly available [2, 6, 13–12, 16, 19, 22]. Yet, only a few are provided as web services. A limited number of algorithms with respective reference data have been published as offline tools, often exceeding the infrastructure and informatics knowledge of diagnostic laboratories [12, 13]. No single one of them is capable of running entirely on local low-cost, low-resource devices. Moreover, large quantities of publicly available datasets, e.g., on Gene Expression Omnibus (GEO) and The Cancer Genome Atlas (TCGA), have not been included as reference data. Diagnostic institutions with high caseloads require straightforward mechanisms to include their own reference data without the need for re-training and re-validation inherent to supervised machine learning. Collectively, these shortcomings significantly impede the clinical utility of available methylation and copy number reference data.

We set out to explicitly incorporate an unsupervised machine learning system and copy number profiler into our diagnostic routine that combines several modes of data acquisition to facilitate integrated diagnoses in surgical (neuro-)pathology. While we absolutely advocate using the existing supervised classification algorithms, we likewise strongly suggest parallel examination of diagnostic methylomes by unsupervised data matching. In support of this notion, Aldape and colleagues recently reported that supervised brain tumour classification renders high-confidence matching scores in only about 2⁄3 of cases [32]. Here, we benchmarked UMAP plotting against a published random forest brain tumour classifier [2]. The result corroborates the clinical utility of our tool across a wide range of human neoplasias and healthy tissues. UMAP further introduces a novel quality criterion for microarray-based methylation profiles and identifies significant divergence of epigenetic profiles across many tumour cell lines.

As defined by the WHO [30], methylation profiling is currently mandatory for the diagnosis of certain brain tumour types. To facilitate methylome-based tumour diagnostics, we designed EpiDiP/NanoDiP to address multiple clinical scenarios including the related global economic challenges for epigenetic and copy number analysis. Our public web service EpiDiP performs unsupervised machine learning and copy number plotting for microarray data free of charge [1, 8, 9, 21]. EpiDiP is a website frontend to NanoDiP, a portable edge computing approach for laboratory integration on cost-effective hardware, widely available, and energy-efficient computer hardware, in particular systems-on-chip/systems-on-module (SoC) and cryptocurrency miners (CCM), addressing recent concerns about global applicability [11]. Our ultra-fast setup eliminates the need for data transfer, network access, high-performance computing clusters, and dedicated computer housing. It also simplifies the assembly of reference data collections. A nanopore sequencing-compatible ambient temperature sample shipment protocol widens the clinical utility of our approach by allowing remote diagnostic institutions to benefit from fast tumour profiling. As a proof-of-concept, our system, which has already been implemented in two foreign diagnostic laboratories, allows molecular tumour classification even before FFPE-based H&E histology is available. Of note, EpiDiP/NanoDiP enables resource-efficient methylome and copy number analyses according to WHO guidelines also in low- and middle-income regions, including those without access to expensive technologies such as immunohistochemistry, microarrays, or conventional sequencing.

Here, we describe the underlying algorithms, strategies of computational optimization, clinical applicability, and diagnostic performance of EpiDiP and NanoDiP in our surgical (neuro)pathology practice.

## Materials and methods

### DNA extraction

Diagnostic biopsy specimens submitted to our institution were routinely examined in the course of intraoperative consultations (frozen sections), followed by standard formalin-fixation and paraffin-embedding (FFPE) for histological workup in most cases. External cases were either received as FFPE blocks or as near-native tumour tissue submitted in SurePath^®^ (Becton Dickinson, USA) or ThinPrep^®^ preservatives (Hologic Inc., USA). For cryopreserved biopsies, one to four 70 μm thick cryosections were collected. For FFPE blocks, a series of 8 or 14 serial sections (4 μm) were collected on glass slides, and an additional H&E section in the middle of each series was used to verify tumour cell content. In samples containing significant non-neoplastic or necrotic areas on the cut surface, viable tumour cell-rich areas were manually microdissected. For native specimens, DNA was extracted with automated commercial systems: Promega Maxwell^®^ FFPE DNA extraction, Promega Maxwell^®^ blood DNA extraction, and Qiagen DNeasy^®^ Blood and Tissue kits on Maxwell or QiaCube^®^ instruments (Promega, USA; Qiagen, Germany). DNA from FFPE specimens was extracted with the Promega Maxwell^®^ FFPE DNA extraction, Qiagen DNeasy^®^, and RecoverAll^®^ kits. Except for differences in average read lengths in nanopore sequencing runs for native materials, all of the above-mentioned kits produced technically valid results.

### Copy number profiling

#### Microarray

Copy number levels were computed and plotted as PDF with Conumee [29]. Detailed version information and installation instructions are included in the NanoDiP repository (Suppl. file 1). In addition, copy number data were stored in binary format to facilitate ad-hoc in-depth plotting and re-annotation for specific loci using plotly [https://plotly.com/]. The latter functionality is accessible through the NanoDiP web interface.

#### Nanopore

Sequencing reads were basecalled with guppy (ONT) and aligned against the human genome (hg19) with minimap2 [18] (hg19 link and version-pinned software in repository). A target read count of 30 reads per bin was defined. Bin size was adapted to the overall read number and read counts per bin plotted (NanoDiP subroutine in Python 3.7).

### Methylation detection

#### Microarray

DNA was bisulfite converted (Zymo research lightning kit) and microarrays (Illumina Infinium Human Methylation beadchip EPIC / 850 K) were processed according to the manufacturer’s protocol (service provided by Life & Brain GmbH, Bonn, Germany; FFPE restoration kit was not applied). IDAT data were processed with minfi and SWAN normalisation [7] as described [10, 11, 23]. The source code is available as part of the EpiDiP software [https://github.com/neuropathbasel/epidip]. In short, a sex chromosome-depleted, blacklist-filtered [4] overlap set of 400’962 methylation sites covered by the Illumina Infinium Methylation 450 K and EPIC v1 arrays (“overlap CpGs”, oCpGs) were extracted as floating-point numbers and stored in binary files to accelerate data loading. EPIC v2 (950 K) compatibility has been added to the EpiDiP web service based on development versions of minfi and conumee (Supplementary file 1).

#### Nanopore

Nanopore sequencing was carried out on Mk1B (Oxford Nanopore Technologies [ONT], Oxford, UK) sequencers connected to control computers running MinKNOW and a customised MinKNOW API (Suppl. File 1). MinION R9.4.1 flow cells, SQK RBK-004, RAP top-up, and WSH-003/004 kits were used. DNA samples, exclusively from native or alcohol-preserved biopsy specimens were sequentially labelled with molecular barcodes (SQK RBK-004), allowing the consecutive analysis of 12 samples per flow cell without carryover artefacts (all kits from ONT). After each run, flow cells were cleaned (WSH-003/004 with DNAse I from Sigma Aldrich). For refrigerator storage (4 to 8 °C), flow cells were filled with ‘storage buffer’ (ONT). No relevant loss of sequencing quality between runs was noted when running up to 14 samples per flow. New flow cells were submitted to the “flow cell check” protocol (MinKNOW UI). Before each run, a 10-minute sequencing “test run” was initiated through NanoDiP to verify and document the successful digestion of the DNA library digestion from the previous sample, thereby also verifying a functional flow cell state before loading the next specimen. Of note, paraffin-embedded specimens are not compatible with the outlined Nanopore-based workflow.

Detailed software version information and installation instructions are included in the NanoDiP repository (Suppl. File 1). In short, guppy (GPU version, ONT) was the basecaller, minimap2 the aligner [18], and f5c (GPU, version 1) called methylation [8] (technical details and GitHub links to all source code in Suppl. File 1). By default, 150 megabases of valid reads (ONT qscore > = 7) were collected, resulting in 5’000 to 10’000 oCpGs using the automatic run terminator function in NanoDiP.

R10 pore sequencing has been implemented in the NanoDiP software and tested with MinKNOW version 23.07.5 / API version 5.7.2. with Guppy basecalling replaced by Dorado. We have established integration of NanoDiP with ONT’s sequencing and basecalling software on both the aarch64 ORIN AGX 32GB SoC (Nvidia, Inc.) and x86_64 hardware with gpGPUs (Nvidia, Inc.), both running Ubuntu 20.04. R10 protocols are still considered early access by the manufacturer and internal clinical validation is ongoing (Suppl. File 1). Detailed description of the NanoDiP hardware/software platform allowing replication is provided (Suppl. File 1).

#### MethylSeq

Tumour DNA extracts previously examined with Nanopore sequencing and methylation microarrays were sequenced with an enzyme-based parallel methylation sequencing panel, covering approx. 390’000 oCpGs (Twist Bioscience, USA) [26] on an Illumina platform according to the manufacturer’s protocols (Twist Bioscience, USA and Illumina Inc., USA). Sequencing resulted in fastq files that were analysed with the nfcore-methylseq bwa-meth/Methyldackel branch (Suppl. File 1), resulting in tab-separated value bedGraph files. In the resulting bedGraph, counts of methylated (#M) and non-methylated (#N) reads are listed per CpG site together with a rounded methylation ratio. In a Python script, the methylation ratio was recalculated (#M/(#N+#M)) at higher precision and divided by 100 to match the data scale of methylation array output from minfi (numbers between 0 and 1). Next, the ratios of oCpG sites were isolated. oCpGs not covered by the sequencing panel were assigned a methylation value of 0.49 to match microarray datasets. Methylation values were stored in a binary float format (detailed in the microarray section and Suppl. File 1).

### Methylation-based classification through unsupervised machine learning

For microarray data, the 25’000, 50’000, or 75’000 most variably methylated probes are selected by standard deviation-based ranking, excluding non-informative signals from dimension reduction. NanoDiP, which runs as the server back-end on our public EpiDiP server, recalculates the UMAP plot for all tumour methylome datasets (approx. 30’000 at the time of submission) as soon as new microarray files are uploaded. As an alternative, installing and running the NanoDiP software locally (including a preconfigured virtual machine; Suppl. File 1) enables free choice (through the UI) of the number of most differentially methylated microarray probes to be fed into dimension reduction. From nanopore-derived sequencing data, oCpGs are extracted from aligned, methylation-called reads and joined with respective oCpGs of microarray-based reference data without probeset optimization; then UMAP is executed. A software flowchart for UMAP plotting is included in Suppl. File 1.

**Fig. 1:**
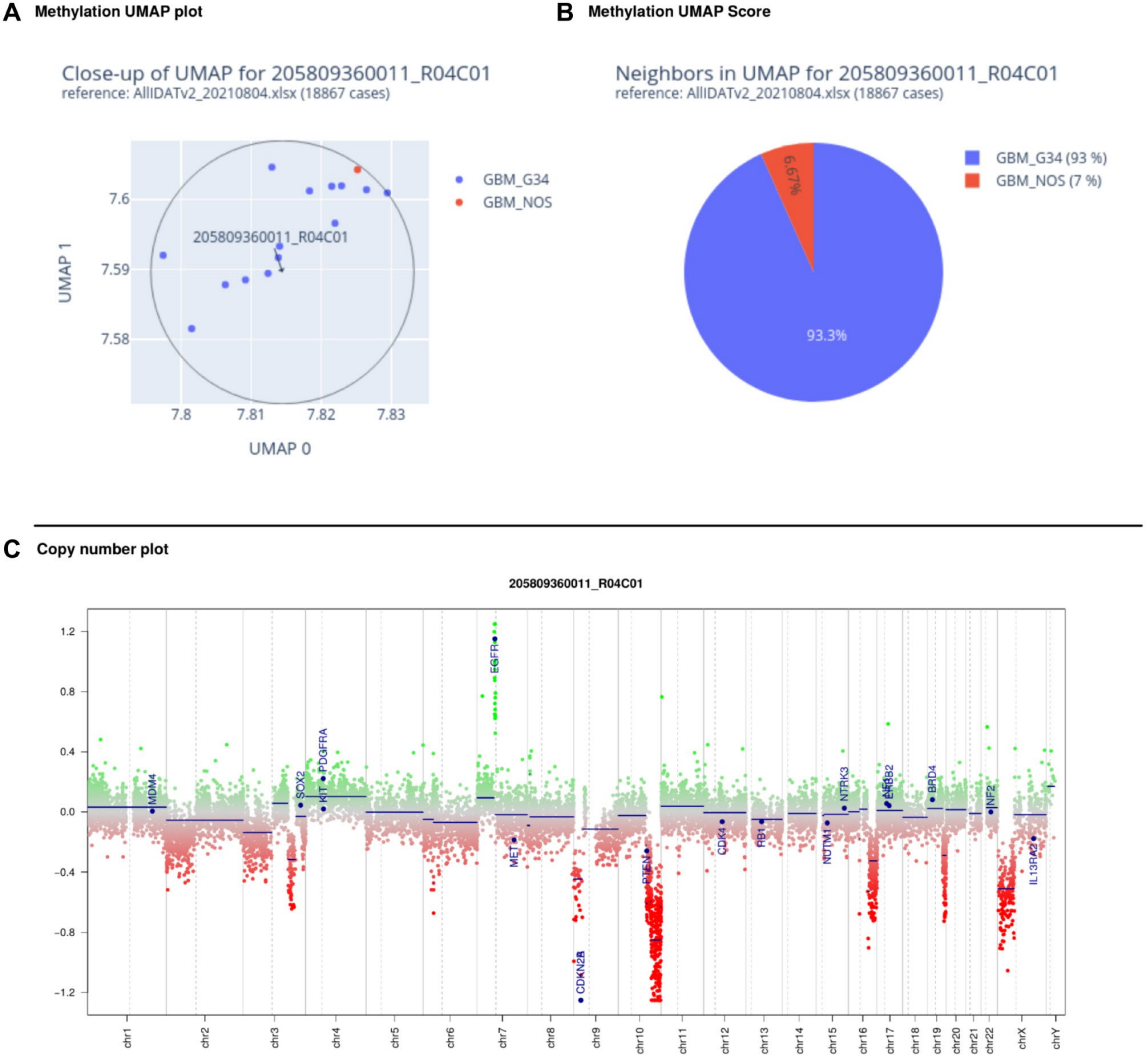
PDF report generated in NanoDiP based on microarray data. (**A**) Zoom in on the UMAP plot to the 15 nearest annotated neighbour cases. (**B**) Annotation counts encountered in A). (**C**) Copy number profile generated with Conumee. The depicted case is a diffuse glioma, H3 p.G34 mutant (CNS WHO grade 4), harbouring an EGFR amplification and homozygous deletion of the CDKN2A/B gene loci

**Fig. 2:**
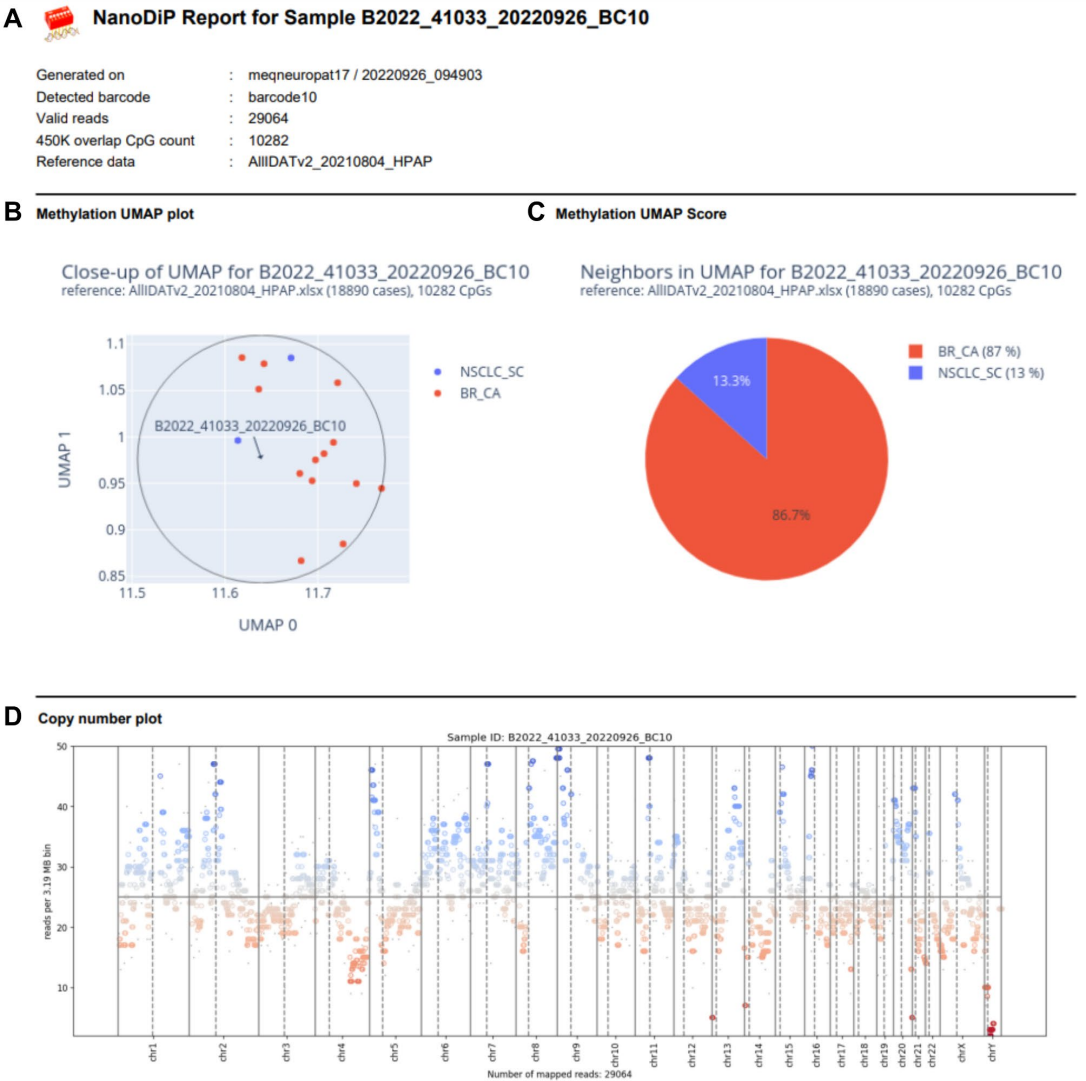
PDF report generated intraoperatively in NanoDiP based on nanopore data from a breast cancer brain metastasis. (**A**) General run information. (**B**) Zoom in on the UMAP plot to the closest 15 annotated neighbour cases. (**C**) Annotation counts encountered. (**D**) Copy number profile generated from read counts

Throughout this manuscript, a Python 3.7 implementation of UMAP (Suppl. File 1) was used. In the reference dataset, at least 8, but typically > 20 cases per entity were present. We therefore considered the closest 15 annotated neighbours in the UMAP plot for classification (Fig. 1A) throughout this manuscript as well as in our in-house diagnostic routine; however, users are free to tune this parameter. The methylation class (MC) for each case was derived from the most abundant MC of the nearest neighbours in the UMAP plot. Besides highlighting the nearest UMAP neighbours, our diagnostic report provides a summary of the entities in pie chart format (Fig. 1B) along with a chromosomal copy number plot (Fig. 1C). Similarly, UMAP scoring and copy number profiling based on nanopore sequencing data are computed. Respective reports can be generated ad hoc during and after nanopore sequencing, allowing preliminary UMAP plot inspection in time-critical situations (Fig. 2).

### UMAP score performance testing

#### UMAP scoring-based brain tumour classification

A subset of 798 cases from our diagnostic routine that resulted in bona fide integrated diagnoses covered by the v11b4 brain tumour classifier reference set GSE90496 [2] were selected for benchmarking.

A series of independent UMAP plots were generated using 50, 100, 500, 1’000, 5’000, 10’000, 15’000, 20’000, 25’000, 50’000, and 75’000 most variably (top) differentially methylated probes (TDMP). TDMPs of 400’962 oCpGs were determined by standard deviation-based ranking [23]. These UMAP plots were generated for datasets in the EpiDiP data lake, mostly derived from TCGA, GEO (including GSE90496, *n* = 2’801), and in-house diagnostic cases (2017 to mid-2022, *n* = 18’433). The resulting plots were overlaid with the brain tumour MC annotation for the 2’801 cases of GSE90496. The remaining cases (*n* = 15’632) were considered non-annotated. The 15 nearest annotated neighbours for each of the 798 test cases were determined and the predominant MC considered the UMAP-derived diagnosis. To better reflect the clinical setting concerning treatment decisions, we simplified the supplied MC annotation of GSE90596 into methylation superclasses (SC). To this end, we grouped, e.g., the various subtypes of glioblastoma, IDH wildtype, into a single superclass (Suppl. File 1). All MC results were translated into SCs. The matching fraction for each TDMP count was summarised. In parallel, the v11b4 brain tumour methylation classifier [2], kindly provided by DKFZ (Heidelberg Germany), was installed locally (R 3.6.3 / x86_64) and executed. For each case, the topmost calibrated score (without cut-off) was considered the v11b4-based diagnosis. Both UMAP-and v11b4-based MCs and SCs were compared to the MCs / SCs representing the integrated diagnosis. Results were counted as either matches or mismatches.

To demonstrate robustness of UMAP plotting, we repeated UMAP plotting on our public EpiDiP platform with a snapshot from Jan. 10th, 2024 (*n* = 31’248 IDAT files, 450 K, 850 K, and 935 K formats). This set includes public uploads since 2019. Details of this benchmarking experiment are provided in Suppl. File 1.

#### UMAP scoring with a pan-cancer dataset

With the introduction of EpiDiP in 2019, our routine application of methylation-based diagnostics expanded into extra-CNS and non-sarcomatous tumour spectra. We have reviewed integrated diagnostic reports for cases not covered by the brain and soft tissue tumour classifiers [2, 16] within the validation cohort obtained since mid-2021. 156 cases were analysed by microarray-based methylation and copy number profiling, e.g., to determine the origin in cancers of unknown primaries, the lineage of lymphomas, melanomas, or to assess MLH1 promoter methylation in various cancers. Of note, only cases in which diagnosis was confirmed irrespective of methylation arrays, e.g. through clinical follow-up or additional molecular testing, were considered.

### DNA read lengths, nanopore sequencing speed

On R9.4.1 flow cells average read lengths typically ranged between 5 and 10 kilobases, irrespective of prior sample preservation in cytology media and mail transfer at ambient temperature. Our in-house process timeline (for details see Suppl. File 1) requires approximately 90 min from tissue arrival to final report. Increased read lengths have led to decreased data acquisition time. Irrespective of read lengths, we have set a threshold of 150 megabases in diagnostic samples which typically reveals 5000 to 10,000 oCpGs. While recently released R10 pore chemistry is supported in our latest NanoDiP release, R10 has (at the time of writing) only been tested with retrospective samples where it enabled classifications comparable to those obtained with R9 pore chemistry (original matched R9/R10/EPIC V1 data from a glioblastoma, IDH-wildtype, included in demonstration VM for evaluation, Suppl. File 1).

## Results

EpiDiP/NanoDiP is an open-source software suite for rapid methylome-based tumour classification and copy number profiling in a standardised manner. Its graphical user interface (GUI) does not require any programming or data analytic skills. In fact, during our in-house routine diagnostics, it is operated solely by laboratory technical staff. Our software can be adjusted to meet laboratory-specific needs through the integrated development environment (IDE). To ensure 24/7 diagnostic availability, EpiDip/NanoDiP runs on robust, resource-efficient, portable hybrid edge computing platforms.

### Graphical frontend facilitates NanoDiP operation

**Fig. 3:**
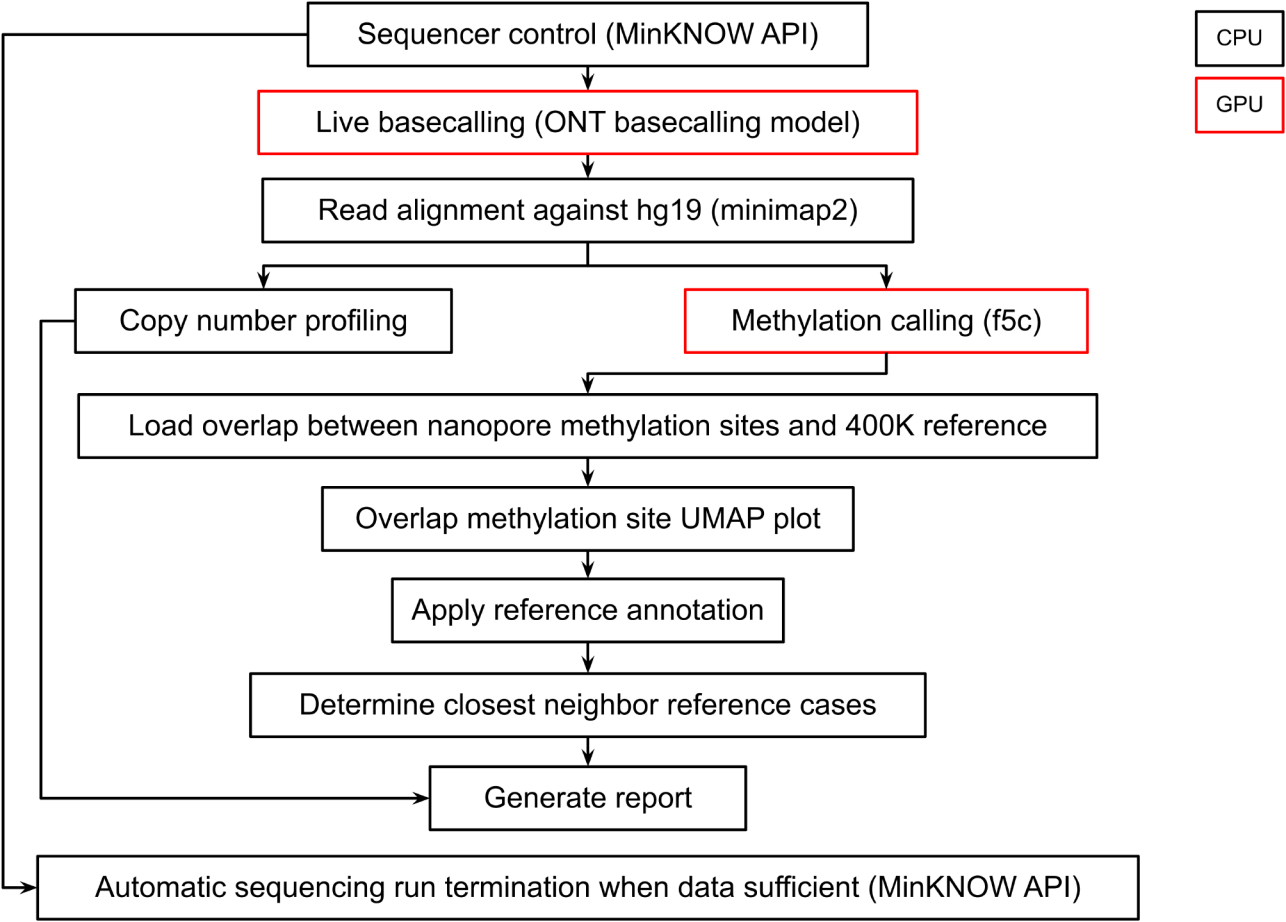
NanoDiP software flow highlighting nanopore sequencer control and data analysis. Computing is shared between CPU (black) and GPU (red) cores depending on memory requirements and the possibility of making use of parallel programming

NanoDiP has a web browser-based GUI (Suppl. File 1, user interface section) for the initiation of sequencing, flow cell integrity check, automatic run termination, and detailed logging. The GUI incorporates system status parameters, a laboratory information system for sample management, and sequencer control. In addition, it comprises live and *post hoc* data analysis including visualisation, copy number plotters for nanopore and microarray data with user-definable gene annotations, and UMAP plotters for nanopore, microarray, and 2nd generation methylation sequencing data. Static PDF reports as well as interactive plots for in-depth analysis of nearest neighbours (e.g. exploration of their copy number profiles) can be generated during and after sequencing (Figs. 2 and 3). In UMAP plots exported as interactive HTML files, copy number plots of reference samples can be readily obtained from our EpiDiP website by clicking on the respective reference case. For advanced data privacy, respective copy number plots can alternatively reside on local storage systems or internal web servers, eliminating the need for an internet connection. The microarray-centred functionality of NanoDiP is also available through our public web service EpiDiP.

### Low-coverage nanopore sequencing reveals diagnostic copy number profiles

**Fig. 4:**
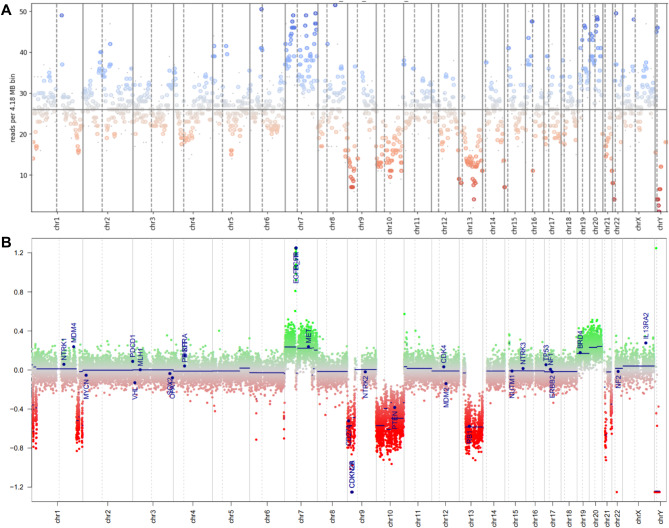
CNV plots of a Glioblastoma, IDH wildtype (CNS WHO grade 4), subtype RTK II. Note the ‘gain 7 / LOH 10’ signature and additional chromosomal alterations that can be appreciated from both the nanopore (**A**) and the corresponding microarray plots (**B**). Circumscribed alterations such as the EGFR gene amplification and complete CDKN2A/B deletion are frequently missed in the nanopore data, precluding meaningful single gene annotations in low-coverage sequencing-based copy number plots

Copy number profiling through binning and read counting revealed copy number plots in all examined specimens with bin sizes between 1 and 10 Mb (Fig. 4A). However, this coverage is too low to identify single-gene amplifications or deletions which are readily visible in microarray data (Fig. 4B). Nevertheless, arm-level alterations (such as LOH 1p/19q, + 7/-10 glioblastoma signatures, 9p deletions, and LOH 22q) are readily detectable at this resolution (Fig. 4A) even with long read lengths and consecutively low read counts, providing an additional layer for integrated diagnostics.

### Match between methylSeq and microarray methylation profiles

NanoDiP enables direct result comparison from different methylation analysis strategies. In a proof-of-concept experiment, MethySeq results were compared with microarray methylation profiles. An interactive UMAP plot for comparison is suitable for detecting concordance or discrepancy between different methylome analysis strategies. We found matching MC determination with the integrated diagnosis for all examined cases (7/7; 100%). The close distances between the seven matching dataset pairs can be appreciated in an interactive UMAP plot (see Suppl. File 1). Thus, methylSeq qualifies as a veritable alternative to microarrays.

### Methylation-based classification through UMAP reaches diagnostic precision

**Fig. 5:**
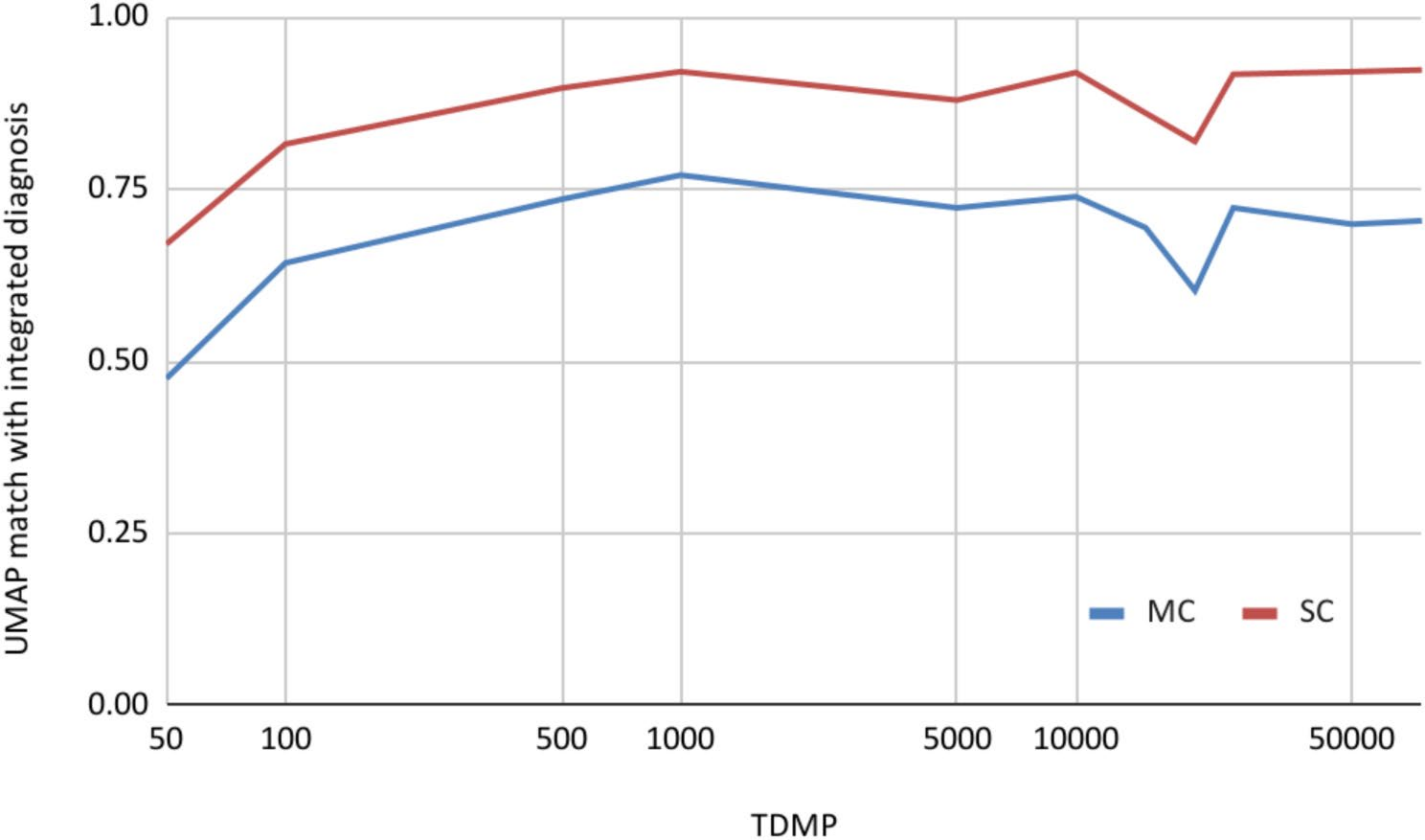
Performance of an unsupervised pan-cancer dimension reduction (UMAP) with partial, brain tumour-restricted reference annotation (GSE90496) against *bona fide* integrated diagnoses (based on clinical, histological, copy number, and sequencing data). 798 routine diagnostic cases were analysed. Between 50 and 75’000 most variably methylated array probes were considered (TDMP = top differentially methylated probes)

A selection of 798 routine diagnostic cases with *bona fide* integrated diagnoses was analysed with the v11b4 brain tumour classifier [2]. The v11b4 classifier reached a consensus of 87% (694/798) with the integrated diagnosis at the MC level and 98% (780/798) at the SC level. Despite large numbers of unannotated datasets in the plot, UMAP scoring of the 15 nearest annotated GSE90496 cases matched the integrated diagnoses in up to 77% (615/798) when considering 1’000 TDMP at MC level and 92% (737/798) at SC level with 75’000 TDMP (Fig. 5). When considering between 10’000 and 75’000 TDMPs, the average consensus was 69% for MCs and 89% for SCs. For each annotation, the plot mostly contained unannotated datasets (15’632/18’433; 84%) besides the analysed case (1/18’433) and GSE90469 (2’801/18’433; 15%). Despite the large fraction of non-annotated cases in this experiment, diagnostic precision remained stable at a high level. Classification accuracy for brain tumours remains stable at 0.8 (MC match) and 0.95 (SC match) when iterating over sets with random removal of 300, 3’000, and 11’000 from the present total of 32’148 cases (Suppl. File 1).

### UMAP addresses a wide spectrum of methylation patterns

**Fig. 6:**
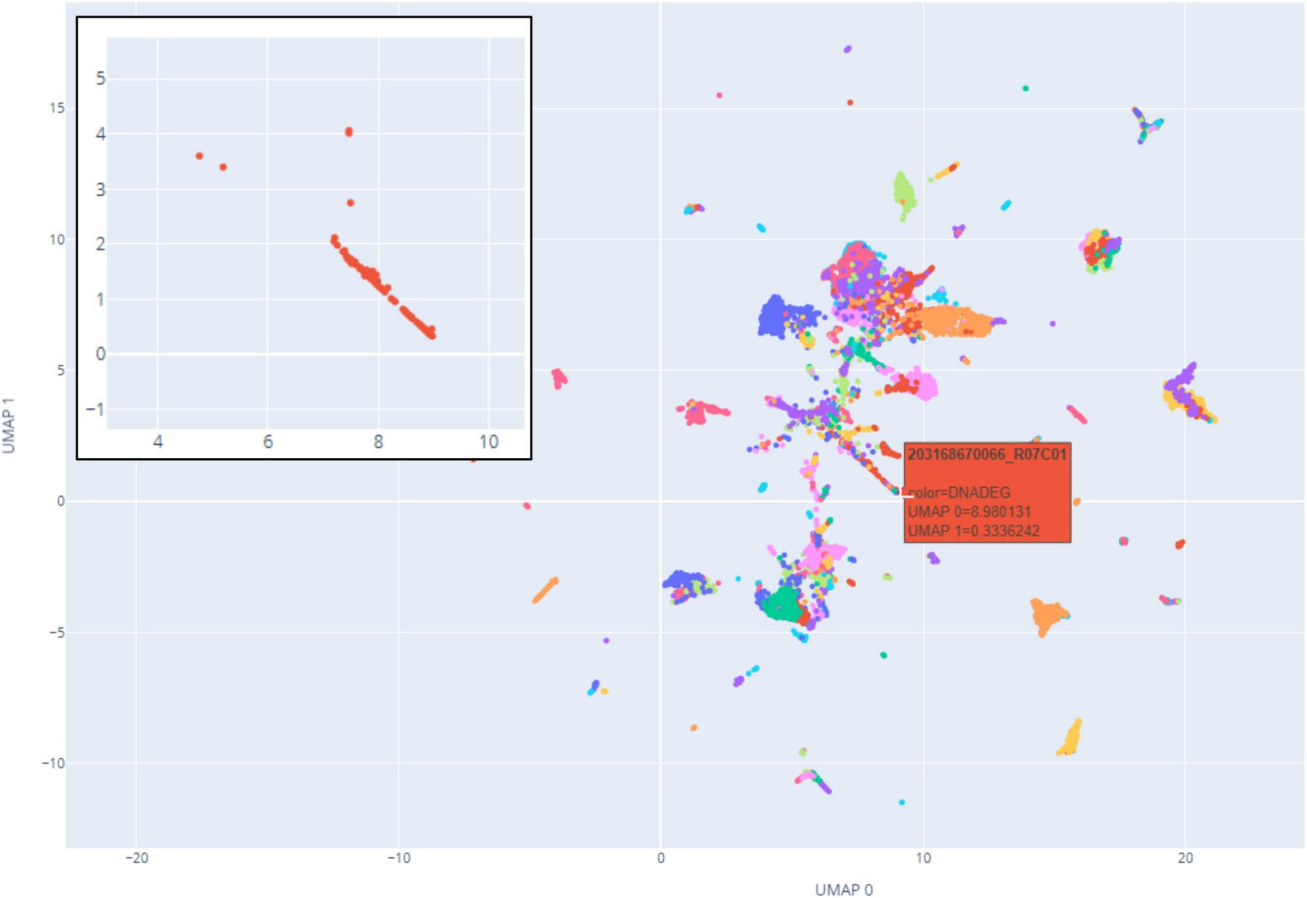
UMAP dimension reduction reveals an alternative microarray quality assessment parameter for methylation data: Abnormal methylation microarray signatures cluster in pan-cancer UMAP plots. Such datasets were annotated as DNADEG. Incoming samples having mostly DNADEG-annotated reference cases within their 15 nearest neighbours should be excluded from data interpretation. The inset (upper left) reveals a tight clustering of DNADEG cases. The red box is an example of a pop-up-on-mouse-hover annotation, revealing at a glance the microarray ID and annotation for each dot in the plot

When examining methylation array data in UMAP plots, distinct clusters became apparent that share a low signal-to-noise ratio in their copy number profiles. Annotation of such cases with the novel MC label (“degraded DNA, DNADEG”) serves as a robust quality control criterion in our institute (Fig. 6). Cases matching the DNADEG pattern were rejected from further data interpretation with pre-trained models [2]. Such samples were subsequently annotated as “DNADEG”.

**Fig. 7:**
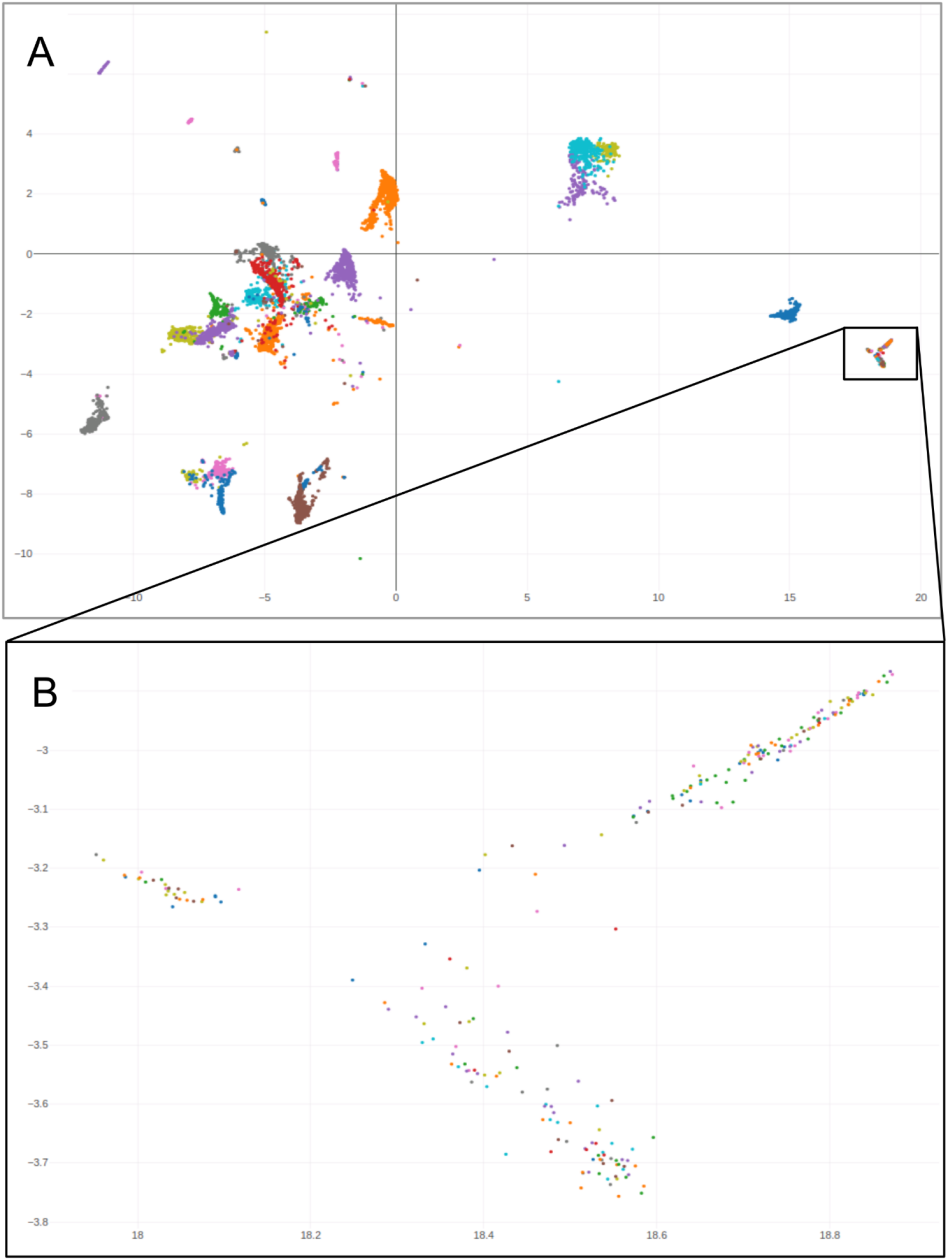
Public pan-cancer UMAP plot highlighting the loss of their original epigenetic signatures. (**A**) Tumour cell line reference datasets cluster together (100% of cases). Note that the cell line cluster lies apart from the respective tumour of origin clusters. Axes: UMAP 0,1. (**B**) Subplot magnification reveals an absence of tumour entity-specific clustering. Cell lines coloured according to their origin, comprising adrenal carcinoma, urothelial carcinoma, breast cancer, cervical squamous cell cancer, colon cancer, diffuse large B cell lymphoma, endometrial cancer, oesophagal cancer, glioblastoma IDH wildtype MES/RTK I/RTK II, hepatocellular carcinoma, cutaneous melanoma, uveal melanoma, mesothelioma, lung adenocarcinoma, lung squamous cell carcinoma, ovarian cancer, pancreatic cancer, extra-adrenal paraganglioma, renal cell cancer, sarcomas, stomach cancer, germ cell tumours, renal cell cancers, and thymomas. An in-depth examination of the plot with regard to annotations and copy number profile visualisation is available at www.epidip.org and epidip.usb.ch

Additionally, the dimension reduction suggests a loss of cancer type-specific epigenetic signatures in cell lines (Fig. 7): When examining UMAP plots containing TCGA methylation data as well as microarray files from GEO, we observed that many of the published cell line methylation datasets cluster together. However, they form clusters apart from their tumours of origin (see www.epidip.org, *_cellc annotations). This finding may have important implications for cell culture-derived epigenetic data interpretation.

### UMAP plotting contributes to non-brain/non-sarcoma cancer diagnostics

We analysed 156 validation tumour biopsy specimens, mostly carcinoma metastases and primary tumours, currently not covered by either brain tumour or sarcoma reference datasets. In approximately 80% (*n* = 126) of these specimens, the UMAP-based data interpretation would have significantly contributed to the integrated diagnosis. Clinically relevant misinterpretation would have been rare 1.3% (*n* = 2), consisting in a methylation pattern that grouped with other *bona fide* reference data but which clearly did not match the clinical situation, e.g. in highly undifferentiated cancers of clinically or molecularly known types. 3.8% of specimens (*n* = 6) aligned with inflammatory and reactive signatures which made it impossible to determine the cancer’s origin. 7.1% (*n* = 11) of cases showed signs of degraded DNA or too low amounts of input material and aligned with MC DNADEG. Another 7.1% (*n* = 11) cases showed methylation profiles not matching with established reference methylation datasets and hence remained uninterpretable. In sum, UMAP often provides essential diagnostic information for neoplasias outside of pre-trained tumour spectra.

### NanoDiP rapidly obtains and translates nanopore data into clinically relevant diagnoses

Following the introduction of diagnostic nanopore sequencing based on random forest classifiers in 2019 [6, 17], we started to routinely record between 100 and gradually increased this to 150 megabases of genomic DNA reads. Since 2021, NanoDiP has been used to control sequencing, classify tumours by UMAP scoring, and generate copy number profiles [11]. Preliminary integrated diagnoses were formulated from cryo-histology and nanopore data. From 493 runs, 377 had *bona fide* integrated diagnoses covered by our NanoDiP pan-cancer reference data. For these 377 cases, UMAP scores were compared to integrated diagnoses derived from histomorphology and microarray-derived methylomes. In 269 cases (71%) the UMAP-based diagnosis remained identical to the integrated diagnosis. When considering clinically relevant methylation SCs instead, a match occurred in 313 cases (83%). Most remaining mismatches were due to insufficient tumour cell content as judged from the low amplitudes of chromosomal copy number profiles and/or tumour methylation profiles masked by pronounced inflammatory changes. Nevertheless, in the majority of cases, nanopore sequencing provided important molecular information in an ultra-fast manner, streamlining subsequent diagnostic workup.

## Discussion

### Clinical utility

The development of EpiDiP/NanoDiP was motivated by the frequent diagnostic challenge in which tumours cannot readily be assigned to defined types using available supervised classifiers [32]. Moreover, for a wide range of neoplasms such as brain metastases or hematolymphoid malignancies, no comprehensive supervised classifiers, including their training data, have yet been made publicly available. In such situations, unsupervised DNA methylation data clustering may provide valuable diagnostic guidance. In contrast, we consider supervised approaches superior for differentiating methylation sub-classes such as medulloblastoma G3 versus G4, or meningiomas [22, 31]. However, supervised classifier scores come with the inherent risk in data interpretation, that there will either be a match for one of the trained (enforced) entities or not. Importantly, in the appropriate clinical context, a cerebellar small-blue-round-cell neoplasm might also represent the metastasis of small-cell lung cancer. As unsupervised machine learning enables a more holistic view of the digital methylome landscape, such challenging scenarios would be easily and quickly detected through the proposed layered approach. Specifically, the positioning of each case in the proximity of similar specimens often assists in narrowing down the list of plausible differential diagnoses. Even unannotated cases with only a clinical description or local reference to previously examined patients might pop up on a UMAP plot, hinting at potential similarities. Therefore, whenever possible, the use of available supervised classifiers should be complemented by unsupervised data clustering. In any case, diagnostic decisions should be made by (neuro-)pathologists, who need to adjust their individual confidence levels for the various methylation analysis approaches. In scenarios, where supervised classifiers or statically annotated reference sets do not converge on a histologically/genetically conclusive diagnosis, unsupervised comparison with semi-annotated cases oftentimes hints at additional aspects to be worked up diagnostically, also enabling potential discovery of novel entities.

Diagnostic (neuro-)pathologists are routinely confronted with the clinically relevant task of matching metastases to potential primary tumours. Often, similarities in histological patterns and targeted sequencing data are considered evidence of clonal relationships. However, in situations of multiple pulmonary masses, it might be relevant to differentiate between independent primaries and systemic metastatic disease. In addition, many driver variants in lung cancers can be shared by other entities, so their presence would be insufficient to prove clonal relationships. Many lung cancers, e.g., exhibit a plethora of traceable, private copy number alterations. In such situations, combined methylation and copy number profiling may not only be more cost-effective but also more comprehensive than targeted sequencing.

It is important to note that a number of diagnostically challenging tumours subjected to methylation profiling are likely to remain currently unclassifiable. Typical warning signs that prompt for cautious interpretation of UMAP results are the location of a case in between two or more reference case clusters and mismatches between present and expected copy number changes for the epigenetic tumour type. With EpiDiP, such cases can be queried against our public resource platform which at the time of this writing comprises some 30’000 methylome datasets. In some instances, such unclear cases cluster together upon dimension reduction and may even share further similarities such as copy number variants. In any case, efforts to match available clinical information can be helpful in identifying what we call ‘digital twin tumours’, and in informing further molecular workup.

NanoDiP allows examiners to define custom reference datasets for UMAP plotting. While this feature facilitates fast incorporation of novel entities, careful use in diagnostic settings is advised, starting with independent in-house reference and validation datasets. If, e.g. an uploaded tumour type is represented by less than 15 reference cases, this needs to be considered when interpreting nearest neighbour pie charts. Furthermore, UMAP tends to cluster one-of-a-kind datasets to the best matching methylation class rather than placing them into unpopulated areas of the plot, which may lead to misclassification. While histological examination in combination with supervised classifiers significantly augments clinical diagnosis [32], UMAP analysis may further improve the interpretability of classifier results although it does not provide confidence scores. This, in particular, holds true for the ambiguous DNA methylation signatures which are often observed as a technical hindrance or due to insufficient DNA preservation. Such samples can be identified based on their similarity to the DNADEG methylation class. Furthermore, distinct copy number alterations such as EGFR or ERBB2 gene amplification could be ascertained by inspecting copy number profiles in conjunction with morphology, revealing both a high likelihood for a particular tumour type and potentially providing additional predictive information. Our approach of enabling examiners to annotate genes and loci of interest through a graphical user interface has - to our knowledge - not been implemented in any open-source methylation and copy number profiling tool (can be evaluated in demonstration VM, Suppl. File 1). Lastly, histologically unclear lesions, where differentials, e.g., include pure reactive changes vs. tumours masked by inflammation, can also be differentiated with high probability based on the presence or absence of chromosomal copy number changes [3, 6, 17].

Lastly, the epigenomic difference between tumour biopsies and derived cell lines across a large spectrum of human neoplasia is not novel [21]. Nevertheless, this striking functional alteration occurring rather uniformly across cell lines of different tissue origins should warrant a critical interpretation of epigenetic data from ex vivo cell cultures and could be monitored by tracking epigenetic drift across cultural passages, e.g. with NanoDiP.

### Global applicability

Native tissues may be preserved in cytological preservatives (see Supplementary file 1), for sample shipment at ambient temperature by regular mail. This approach enables fast methylation and copy number profiling in settings lacking NanoDiP infrastructure. Storage of native tissue in standard cytology preservatives for up to 21 days did not impair methylome analysis. NanoDiP can guide diagnostic and patient management decision-making, thereby reducing laborious histopathological as well as immunohistochemical workup and potentially even guiding targeted sequencing. Therefore, the nanopore-based strategy may significantly lower diagnostic costs [6, 17]. Even with shipping time included, tumour classification from remote is possible before FFPE-based histology and immunohistochemistry are available to local pathologists.

**Fig. 8:**
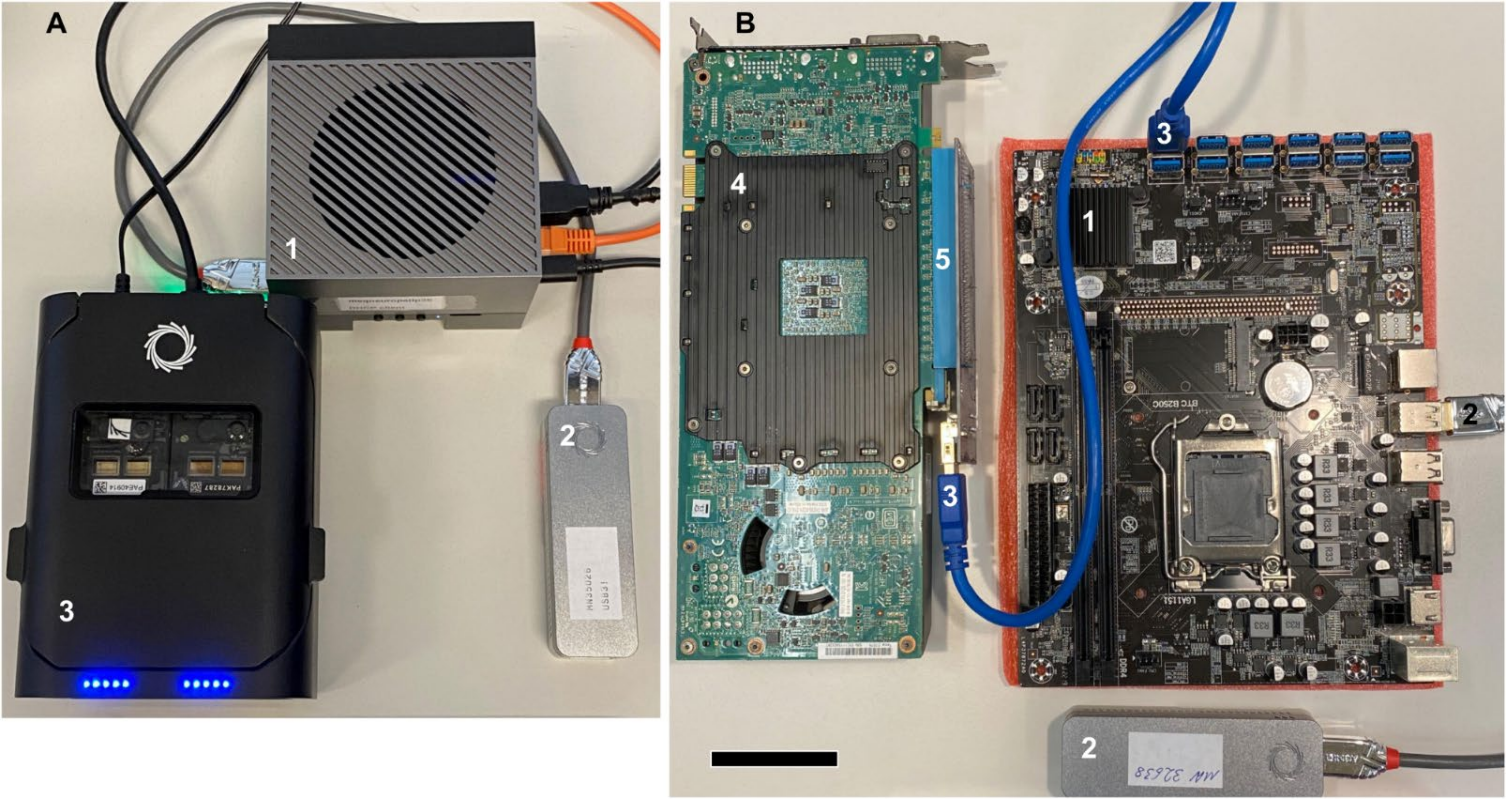
Cost- and space-effective, energy-efficient and portable edge computers for NanoDiP. **A**: Nvidia ORIN CPU/GPU hybrid SoC 32GB developer kit (1) with USB3-attached Mk1B (2) and P2 Solo (3) sequencers. **B**: Minimalistic cryptocurrency mining mainboard (1) with attached Mk1B sequencer (2) and multiple PCIe connectors in robust USB3 plug format (3) to attach one or more gpGPUs (4) and NVMe modules through riser boards (5) for up-scaling. CPU, RAM, NVMe, and power supply were removed to improve visibility. Components are designed to be mounted on open frames. Scale bar: 5 cm

Edge computing concepts [25, 27] were central in the design of NanoDiP. They are reflected by code optimization to run on financially attractive, long-term supported CPU/GPU hybrid SoCs (Fig. 8A). NanoDiP provides a user-friendly graphical interface for controlling sequencing and data analysis. While, in urgent situations, preliminary data examination is already possible during sequencing runs, final tumour classification is rapidly obtained upon completion of sequencing. The edge computing concept in the diagnostic laboratory eliminates the need for data transfer and thereby avoids potential patient privacy and security challenges. As an offline system, NanoDiP can run in mission-critical settings in the absence of networking resources. As power requirements for SoCs are low (approx. 50 W), a battery power source would enable sequencing and data analysis. As an alternative to SoCs, particularly in countries with import restrictions on CPU/GPU SoCs (potential military use), widely available CCM hardware is an equally cost-effective alternative (Fig. 8B). To make a proof-of-concept to our statement, the more performant one of our two public EpiDiP mirror sites (epidip.usb.ch) runs on CCM hardware. While not as energy-efficient as SoCs, CCMs also feature a low power consumption (due to their intended purpose) while idling (< 100 W) and do not require hosting in a computing centre. The majority of available gpGPUs can be mounted on so-called mining rigs due to highly flexible power supplies and the absence of physical constraints. The sole limitation of these robust and energy-efficient mining mainboards is memory capacity. However, most CCMs support 32GB RAM, sufficient to run all features of NanoDiP. In sum, the edge computing-based NanoDiP concept decreases the time from sample receipt to result and can be set up in most institutions at a low cost. This enables building up own, private reference data lakes from sources of choice, e.g. in-house specimens or public repositories like GEO.

With NanoDiP, nanopore sequencing data are acquired in a standardised manner, base-called, mapped to a human reference genome, methylation-called, methylation-profiled, and copy-number-plotted on a standalone computer. By avoiding the frequently updated (i.e., changing) UI provided by ONT and instead controlling the sequencing through the MinKNOW API, our workflow is specifically adjusted for autonomous operation by laboratory technicians. The option of automated sequencing run termination upon reaching a defined number of high-quality bases makes NanoDiP a walk-away solution (recently adopted by ONT). By defining a reference dataset, tumour typing relies on selected microarray methylation information of *bona fide* reference cases, all in parallel on the same computer. No data transfer to an external computer infrastructure is required, ensuring the highest possible data confidentiality. NanoDiP directly provides the examiner with interactive plots and interpretable reports in the context of pan-cancer or subgroup-specific reference datasets. The possibility to choose between different annotated reference datasets and visualise unclassified data in the context of annotated cases makes NanoDiP well-suited as an assistive system rather than a fully automated diagnosis-generating tool. For increased security and stability we advocate for offline use by running NanoDiP behind a firewall on separate routing hardware/software (we use routers running OpenWrt) with network access restricted to the local intranet for institution-internal backups, export of reports, and transfer of sequencing data to alternative workflows such as NanoDx [17] or Sturgeon [28].

Diagnosticians do not necessarily require extensive (bio)informatics knowledge to interpret the data which, as opposed to supervised machine learning (ML) approaches, leaves room for interpretation within clinical, radiological, and histopathological contexts. Our software is provided as open-source. Therefore, with minimal effort, institution-specific reference datasets can be assembled and used for the interpretation of many (tumour/tissue) spectra. Adding software features is facilitated through a Jupyter Notebook IDE. For laboratories solely relying on microarray data, our public EpiDiP service and the offline UMAP plotter in NanoDiP provide freedom of data interpretation not offered by supervised ML systems. Our approach establishes a direct sample-to-tumour-subtype classification on financially attractive edge computers, with as low as ∼ 5% of the electric power needed relative to a high-performance computing infrastructure. Of note, unsupervised UMAP plotting does not require a training process or specific adjustment prior to use for classification. UMAP is computationally lightweight and GPU-augmentation has been established, therefore being a prime choice for edge computing approaches despite the fact that alternative, potentially more accurate approaches exist. In sum, NanoDiP runs entirely on low-power, low-cost systems as opposed to the majority of supervised systems, in particular for their training process [17, 28].

Criticism [20] of the current WHO CNS tumour classification [30] has challenged the central role of methylation and copy number profiling in diagnosing brain tumours. In particular, the case has been made that resource-limited low- and middle-income countries would not readily benefit from scientific advances in the field, since microarray infrastructure for methylome profiling is neither available nor affordable in many geographical regions. Nevertheless, the current WHO CNS tumour classification has defined methylome profiling as an essential criterion for the diagnosis of certain brain tumour types. Both the critical letter [20] and the current WHO classification [30] have not considered the potential of nanopore sequencing [6, 17, 28] as an affordable, mobile molecular data acquisition technology that enables methylation and copy number profiling nearly anywhere in the world. While initial work relied on data analysis with high-performance compute clusters [5], at least for the training process [28], our approach provides an integrated solution to derive methylation-based classification from intraoperative, native biopsies within two hours at minimal cost. The workflow presented here not only runs on affordable computer hardware but also combines sequencing control with data analysis so that neither molecular biological nor computer science expertise is required for operation. The entire setup comprising the computer, datastore, and sequencer has a physical footprint the size of a shoe box (Fig. 8A) and low power consumption, which is key to running it on portable power sources such as batteries, solar panels or fuel-driven generators. Ready-to-use SoC-based NanoDiP platform could hence be mailed to (and installed at) almost any institution. The NanoDiP computer can be operated remotely through an encrypted internet connection, which has been particularly helpful during the establishment phase in institutions without prior experience. A demonstration virtual machine (VM) provided alongside this manuscript (Suppl. file 1) enables colleagues with a working environment to explore its potential benefit despite the hardware limitations of a VM. The VM will significantly facilitate the local installation process.

Our open-source digital pathology resource EpiDiP/NanoDiP highlights the added value of examining unsupervised ML to complement existing supervised ML strategies for defined tumour spectra, optimising integrated diagnoses. Benchmarking against a supervised random forest classifier reveals acceptable precision while widening the diagnostic horizon in an unprecedented manner.

### Limitations

Fast product development cycles with frequent updates in methylome data acquisition software/technology impair harmonised downstream analysis. Despite the fact that efforts towards technology-independent data formats are currently underway, future use of our software will likely require respective adaptations. This is a widely known adverse phenomenon in the field, affecting the applicability of most previously published methylome-diagnostic workflows [5, 6, 8, 9, 12–14, 17, 24, 28] unless they are constantly re-adjusted to respective methodological changes.

### Electronic supplementary material

Below is the link to the electronic supplementary material.

Supplementary Material 1

## Data Availability

A fully functional demonstration instance of NanoDiP in the form of a VirtualBox™ for processing 450 K/EPICV2 methylation array and preprocessed nanopore sequencing data is available through the two EpiDiP mirror sites: https://www.epidip.org, https://epidip.usb.ch. If https is unavailable, http may be used alternatively. The source code along with installation instructions is available at: https://github.com/neuropathbasel/nanodip, https://github.com/neuropathbasel/nanodip_dependencies, https://github.com/neuropathbasel/nanodip_dev (for R10 and EPIC V2), https://github.com/neuropathbasel/epidip (legacy website code), https://github.com/neuropathbasel/methylseqscripts. Herein are contained download mechanisms that will provide access to processed reference data which can be utilised with NanoDiP. A pan-cancer reference data are also included in the demonstration VM (Suppl. File 1). The www.epidip.org and epidip.usb.ch websites process uploaded IDAT microarray data (450 K, EPIC V1 and V2) free of charge and incorporate them in an overarching UMAP plot. EPIC V1 and R9 / R10 Nanopore datasets for testing purposes have been included in the demonstration VM (Suppl. File 1). Raw nanopore datasets from our routine diagnostics may not be shared as they may contain sensitive genetic information. MethylSeq datasets are available from the authors upon reasonable request (large data size). All source code including reference data is provided for non-commercial use only. Usage in diagnostic settings occurs at the sole responsibility of the treating physician.
