# Supplementary material for: EpiDiP/NanoDiP: a versatile unsupervised machine learning edge computing platform for epigenomic tumour diagnostics
Source: Acta Neuropathol Commun. 2024 Apr 4;12:51. doi: 10.1186/s40478-024-01759-2 (PMC10993614; doi:10.1186/s40478-024-01759-2)
Supplement: Supplementary file 1 — Supplementary Material 1 [file 40478_2024_1759_MOESM1_ESM.pdf]

EpiDiP/NanoDiP: Supplementary File

EpiDiP Public Website UMAP Robustness

To demonstrate robustness of our public EpiDiP UMAP plotting tool, we have iteratively re-calculated UMAP plots from a total of 32'148 datasets (450K/850K/935K). We have applied the reference annotation described in the manuscript to a total of 19'967 cases within this set comprising the current publicly available case annotation. We have then restricted this annotation to the brain tumour entities (methylation classes, MC) from the Brain Tumour Classifier v11b4/GSE90596 and also translated them to methylation superclasses (SC) as described in the Methods section. Randomised removal of 300, 3'000, and 11'000 cases from the total of 32'148 cases was performed in 10 iterations each, resulting in 30 randomly reduced sample cohorts (31'848, 29'148, and 21'148 cases, respectively). Each of the 30 randomly reduced case sets was UMAP-plotted three times, resulting in 90 iterations in total. The 15 nearest annotated neighbour scoring system, as described in the manuscript and as available on our EpiDiP website, was applied.

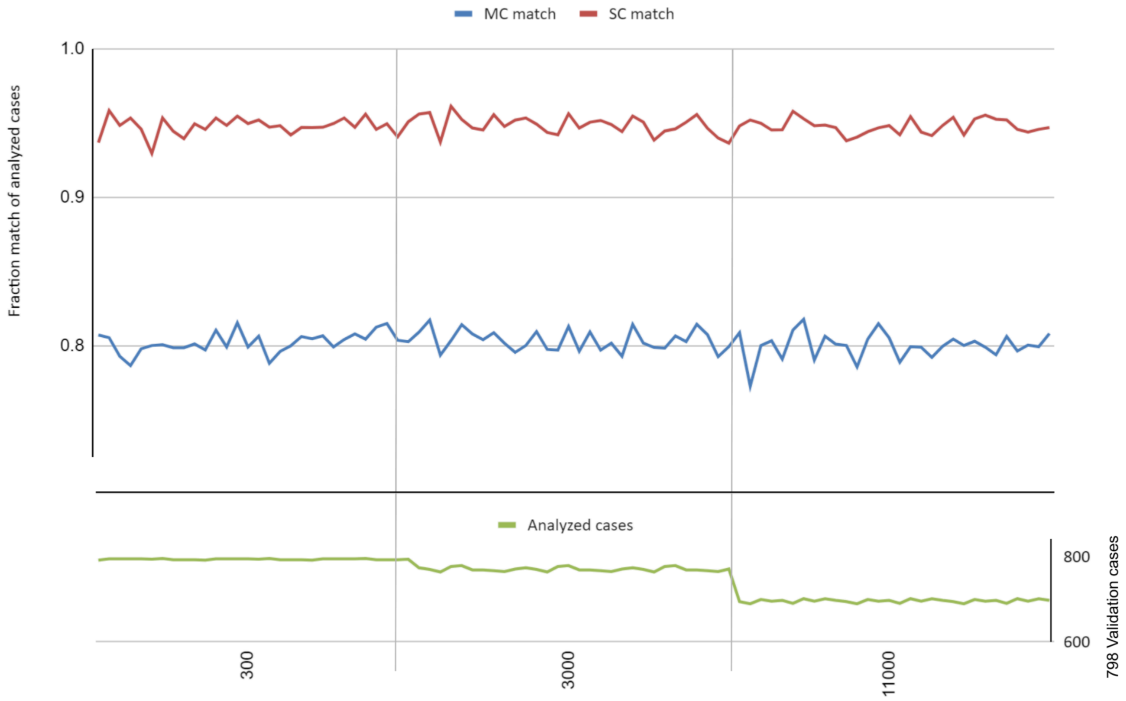

30 UMAP iterations each with 300, 3000, 11000 randomly dropped cases from 31248 total cases

No matter which numbers of cases (300, 3'000, or 11'000) were removed, 15 nearest annotated neighbour classifications were not notably influenced. Note that due to randomised removal of cases, also fractions of validation cases were randomly removed across iterations.

	MC match			SC match		
cases removed	average	median	stddev	average	median	stddev
300	0.8021	0.8018	0.0068	0.9479	0.9484	0.0060
3000	0.8034	0.8021	0.0071	0.9488	0.9493	0.0063
11000	0.7999	0.8000	0.0071	0.9479	0.9476	0.0048

Sample Preservation for Nanopore Sequencing

Cytology-preserved tumour biopsies (SurePath®, Becton Dickinson, USA; ThinPrep®, Hologic Inc., USA) were used to analyse tumours from referring centres outside the Basel area. DNA extraction from cytology-preserved tissue scrolls or effusion cell pellets was performed after washing with tap water. Native biopsies submitted in cytology preservatives were routinely analysed after 1-4 days, corresponding to postal shipment times. For comparative tests, we also stored native tissue in cytology preservatives for up to 21 days prior to analysis. Samples were kept at ambient temperatures; no temperature logging during shipment by regular mail was performed.

Laboratory Process Timeline of NanoDiP

The manual steps to examine a specimen with NanoDiP require several hands-on interventions, detailed in a flow chart.

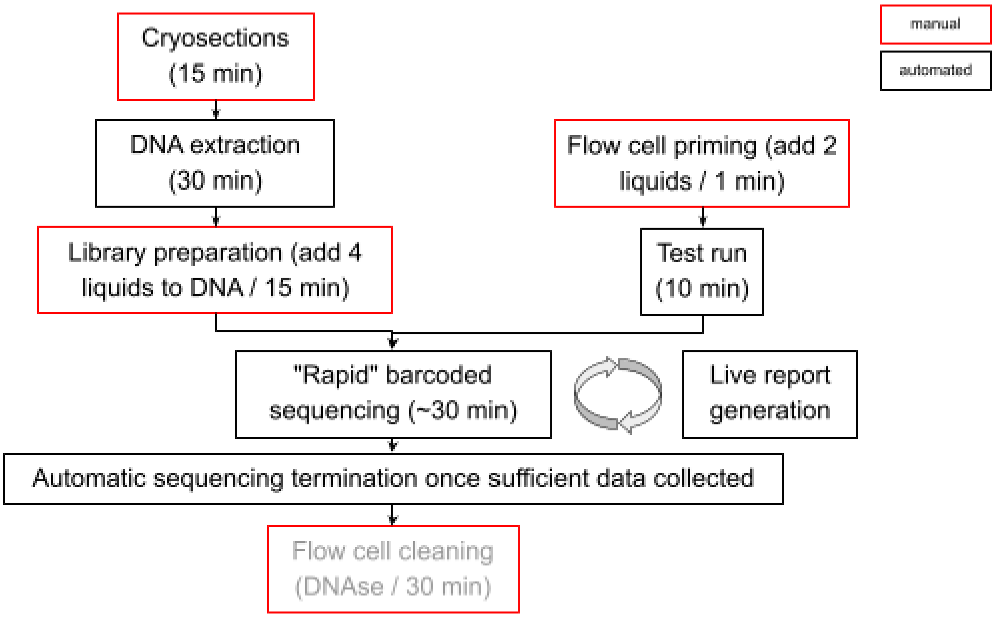

The diagnostic workflow can realistically be completed within 1h 30min, in particular if a fresh sequencing flow cell is used for an urgent specimen.

EpidiP/NanoDiP - Hardware and Software

Local Laboratory Hardware

The code of NanoDiP is open-source and meant to be adjusted to individual laboratory needs (integrated Jupyter Notebook). Hard- and software requirements including hardware costs are detailed in Suppl. File 1. Briefly, NanoDiP is designed to run on aarch64 CPU/GPU hybrid SoCs and gpGPU-equipped x86_64 computers running Linux (Xubuntu 18.04, 20.04), particularly including cost- and energy-efficient CCM hardware. Alternatively, NanoDiP itself runs systems lacking a gpGPU, e.g., virtual machines and high-performance CPU-only compute clusters with the only limitation that 3rd party software (at the time of writing some nanopore basecallers) requiring a GPU can no longer be integrated. The graphical NanoDiP user interface is based on the minimalist web framework CherryPy. Recommended hardware includes >= 8 CPU threads, >= 512 GPU cores, >= 32 GB RAM, >= 1 TB NVMe. For CPU/GPU hybrid SoCs, 32GB shared RAM is sufficient, for PCIe-connected gpGPUs >=8 GPU RAM is suitable.

In our hands, SoCs enable reliable daily routine diagnostics with uptimes of well over 1 year. On SoCs, such as the Nvidia Jetson AGX Xavier 32GB, NanoDiP can control up to three Mk1B sequencers in parallel for GPU-based base and methylation calling, complemented by CPU-based UMAP and copy number analysis (Figure 3). As mentioned, such SoC systems are intended to run without an internet connection and consume approx. 50W for SoC including a storage device. They have a small spatial footprint (Figure 8A in main text).

Public EpiDiP Webserver

We currently provide EpiDiP web services in two instances on x86_64 computers, one of them with gpGPUs. GPU system: CCM with 16 CPU threads, 32 GB RAM, 3 RTX3080 with 10GB RAM each, 16TB hard drive, 4TB RAID1 NVMe. Public rental virtual server: 12 CPU threads, 64GB RAM, 2TB hard drive.

The EpiDiP web frontend consists of an R/shiny-based user interface enabling upload and validation of microarray data files (IDAT), as well as presenting the UMAP in an R/shiny/plotly web application. It additionally links to the microarray-centred portion of the NanoDiP web interface to facilitate PDF report generation based on the current (public) UMAP plot. Lastly, the underlying web server (nginx), provides a pre-configured CPU-only instance of NanoDiP in a virtual machine image for exploratory purposes. The public EpiDiP website is available with and without SSL (https/http) to ensure wide compatibility, particularly for integration in command line-based data analysis workflows.

Hardware Details

The term "edge computing" summarises hardware/software systems at the "network edge", i.e. smaller-scale computers which can be embedded in laboratory equipment such as sequencers, as opposed to high-performance compute clusters requiring dedicated infrastructure rooms (cooling, high-power electricity). We have chosen GPU-augmented edge computers that are widely available at an affordable cost (~EUR 2000). Two major processor platforms, x86_64 and ARMv8, have been evaluated. As an alternative to classical PCs, we have set up several NanoDiP systems with cryptocurrency mining mainboards (e.g., H510 PRO BTC+, Asrock) both on so-called mining rigs (open frames that hold GPUs) and rack-mountable cases. Mining rigs have the advantage of avoiding physical constraints when multiple GPUs are to be mounted, both in the form of dimensions as well as power supply connectors. With an emphasis on their intended purpose, mining mainboards are designed to consume as little power as possible while maximising the PCIe connectivity that we use for gpGPU and NVMe.

More recently, we have evaluated the R10 pore which required significant adaptations of the software portions responsible for obtaining and processing nanopore, previously working with the R9 pore. We are now supporting the widely available ORIN AGX Developer Kit SoC (Nvidia, USA) which is the successor platform of the AGX Xavier. The latter can equally be employed and is currently sold by third-party industry suppliers, including a 64GB RAM version. We have successfully tested two such 64GB AGX Xavier systems (dsboard from forecr.io, Turkey and from Auvidea, Germany). The table below lists hardware including costs for those computers that have been used to perform our retrospective UMAP analysis (Mac Pro A1289) as well as our routine diagnostic systems (AGX Xavier in Basel and Münster, ZBOX in Frankfurt).

**Computer Model<br>GPU Model<br>(year)**	**Mac Pro A1289 (2010)<br>RTX 2070 (2019)**	**ZBOX (2021)<br>RTX 3060 (2021)**	**Jetson AGX Xavier<br>32GB (2021)**
**Computer Type**	2nd hand desktop PC with new GPU	Entertainment PC with GPU	CPU/GPU hybrid SoC developer kit
**Manufacturer**	Apple / Gainward	Zotac	Nvidia
**CPU architecture**	x86_64, Intel Xeon W3530 @ 2.8 GHz	x86_64, Intel i5 i5-10400 @ 2.9 GHz	ARMv8, SoC rev 0 (v8l) @ 2.2 GHz
**CPU threads**	8	12	8
**GPU architecture, bus**	Turing, PCIe	Ampere, PCIe	Volta, SoC
**GPU cores**	2304	3584	512
**System RAM**	32GB	32GB	32GB shared CPU/GPU RAM
**GPU RAM**	8GB	12GB	
**SSD storage**	1TB / Samsung 980 Pro M2 Key on PCIe	1TB / Samsung 980 Pro M2 key on PCIe	1TB / Samsung 980 Pro M2 Key on PCIe
**Computer cost**	~ USD 500, 2nd hand	~ USD 1500	~ USD 800
**Accessory costs**	~ USD 800	~ USD 300	~ USD 150
**Power consumption**	~ 250 W	~ 250 W	~ 25 W

Consumable Costs

Nanopore (ONT) and Infinium Methylation microarrays (Illumina) both in conjunction with DNA extraction (Qiagen) sum up to approx. EUR 190 per sample and analysis (sequencing or array). This holds true if 150

megabases of DNA are sequenced per sample and at least 6 runs are performed per MinION sequencing flow cell with the SQK-RBK004 / RAP Top-up kits. The cost excludes the FFPE restoration kit (Illumina) which is not required for natively extracted DNA and typically not necessary for fresh paraffin blocks.

Software Outline

We chose Ubuntu 18.04 as the operating system for development and production. With the implementation of the R10 pore by ONT, we have switched to Ubuntu 20.04. Closed-source code within our setup is limited to software provided free of charge along with consumables by Oxford Nanopore Technologies (ONT) and the NVIDIA software portions for gpGPU utilisation. The ONT software handles sequencer control and basecalling. It is interfaced through the MinKNOW API, (https://github.com/nanoporetech/minknow_api), a Python API to control sequencer and (to some extent) live basecalling in parallel to the so-called MinKNOW UI application. The MinKNOW UI is a general-purpose, technically limited user interface for the sequencing device. The ONT-supplied basecallers Guppy and (at the time of writing) Dorado use supervised machine learning models that run significantly faster with GPU acceleration than in CPU mode. Therefore, the GPU implementations of guppy were used throughout the project. For R10 support, the current beta version of NanoDiP uses Dorado instead of Guppy for basecalling. This change, enforced by ONT, now requires a gpGPU. The MinKNOW API and portions of MinKNOW are written in Python 3.7 (binary supplied by ONT), hence all development focused on this version of Python. Notably, we have been unable to run the MinKNOW API in Python 3.8. Guppy and MinKNOW binaries are provided by ONT for Ubuntu 16.04-, 18.04-, and 20.04-based computers with x86_64 and ARM processors. The ARM implementation has been adapted from the MinIT, and more recently the Mk1C device distributions (ONT). Both the MinIT and Mk1C devices contain a predecessor of the Nvidia Jetson AGX Xavier CPU/GPU hybrid SoC. Nanopolish and f5c were compiled from the source. We made tested, pinned versions (supporting R9) available through our GitHub repository. Microarray data import requires a multitude of R packages, in particular minfi and Conumee. To ensure reproducibility, we pinned R to version 4.1.1 and Bioconductor to version 3.14 (detailed installation script in NanoDiP repository https://github.com/neuropathbasel/nanodip) for 450K/850K arrays. 935K (EPIC v2) arrays are supported in the current development branch (https://github.com/neuropathbasel/nanodip_dev) through adapted versions of minfi and conumee (links below).

The aarch64 (ARM) platform requires an in-place compilation of R and all dependencies. We provide in-place compilation scripts for aarch64 and x86_64 that enable building NanoDiP from the source on the target computer. All Python dependencies are installed with Pip (R9). The development branch NanoDiP version is supplied as a VirtualBox™ VM.

Jupyter Notebook is the integrated development environment in which we developed our software. Python and shell (bash) knowledge is sufficient to adapt our software to custom needs. NanoDiP is a CherryPy-based web application with a minimalistic, lightweight graphical user interface to initiate nanopore sequencing and launch data analysis during or after a run. Microarray data processing is controlled through the same user interface. Since NanoDiP also represents the core of our public EpiDiP [1–3] web service for dimension reduction plots of methylation microarray data, a local NanoDiP installation enables users to create, curate, and annotate their own reference case collections, eliminating the need to upload their array data outside their institution. All nanopore-based analyses are computed locally. As part of the NanoDiP developer mode through Jupyter Notebook, we provide access to the MinKNOW (R9) playback mode: Previously recorded runs can be recapitulated indefinitely from raw sequencing data, saving on reagents and flow cells during software tests. For productive setups, the Python code exported from Jupyter Notebook is executed as a server application.

The graphical user interface was adapted to the needs of medical-technical staff regarding sequencing run control and of (neuro)pathologists for data analysis and interpretation. Interactive HTML plots (Plotly) provide the (neuro)pathologist with diagnostic information. In addition, static reports in PDF format can be generated for import into compulsory medical documentation systems and clinical reporting. The PDF reporting functionality is also part of the EpiDiP web service. Connection to laboratory information systems is facilitated due to the open source code of NanoDiP, the export of PDF reports, and the possibility of using barcode scanners to register sample IDs. NanoDiP supports headless operation from the command line without reconfiguration (e.g., for benchmarking and research, or to generate and pull reports from the public EpiDiP server in an automated manner). Despite our recommendation to run NanoDiP with GPU support for increased processing speed, we developed NanoDiP GPU-agnostically to run on CPU-only x68_64 or ARMv8 systems including virtual machines as well. High-performance computing platforms which compensate for the lack of a GPU by allocating

many CPU nodes and RAM can also run NanoDiP for data analysis (for R9 data). However, since the Dorado basecaller, unlike the prior Guppy, no longer offers CPU-only operation, R10 nanopore basecalling requires a GPU.

Performance was optimised by using random access binary files residing on PCIe-attached NVMe memory for microarray reference CpG data and making use of gpGPUs for parallel computing tasks. A scheduler avoids hardware overprovisioning.

UMAP Plotting Outline

UMAP plotting occurs after standard deviation-based methylation site selection. This is accelerated by utilising a gpGPU. The figure below illustrates the data flow on the public server as well as in the local instance of NanoDiP. In absence of a CUDA-compatible gpGPU, the system uses numpy instead of cupy functions, i.e. it is possible to run the program without modification, although at a lower speed.

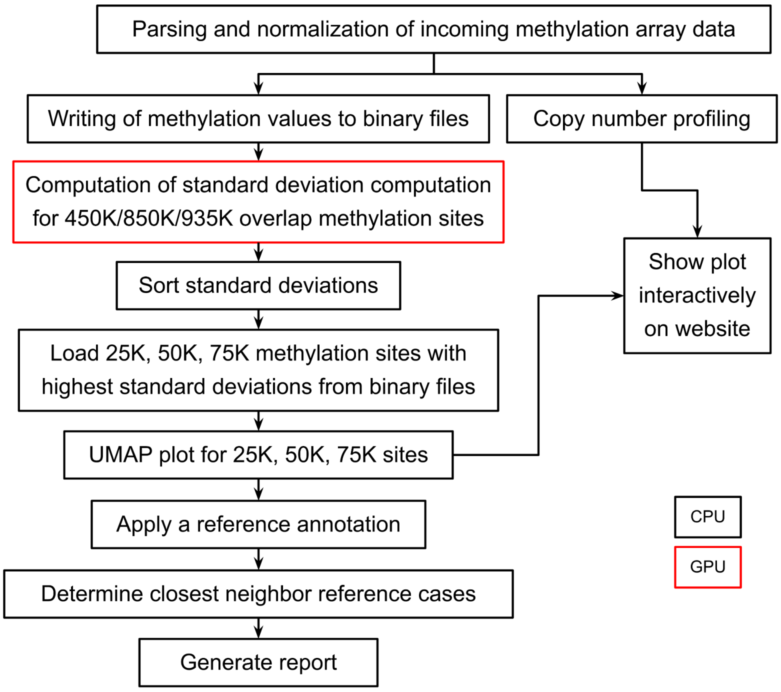

GPUMAP Limitations and Alternatives

An instance of gpumap is - at the time of writing this manuscript - still available as a service since 2019 (legacy page link on www.epidip.org). The gpumap package is no longer maintained by the author since 2019 and has only been tested in CUDA 8, 9, and 10 environments. It depends on faiss (https://pypi.org/project/faiss-gpu/) which is not available for ARM platforms. The current CUDA version 11 does not work with gpumap. As a consequence, we have - in their current versions - restricted EpiDiP and NanoDiP to only use the Python CPU implementation of UMAP (umap-learn) and have instead accelerated standard deviation computation using cupy. Future NanoDiP versions will - again - be able to utilise auxiliary gpGPUs by switching to the RAPIDS AI version of the UMAP python library (Nvidia, inc.). This option is particularly attractive for cryptocurrency mining hardware hosting multiple GPUs.

Edge Computing

Following the general idea that sharing of sequencing data through various networks including the public internet is problematic in a medical care setting and that computational infrastructures may be limited, we have sought to implement NanoDiP in an affordable long-term support industrial SoC in addition to conventional x86_64 PC hardware, particularly cryptocurrency miners that work well with consumer-grade "gaming" gpGPUs. The term "edge computing" refers to bringing the data evaluation to the network "edge", i.e. to process all data right at the place where it is obtained. In terms of laboratory accreditation, but also for software development, well-defined compute platforms that include both CPU and GPU features were chosen. This enables NanoDiP to be provided as a "one-stop shop" and almost "one button" solution so that

(neuro)pathologists and medical technical staff are not required to configure the computer platform themselves. Rather, NanoDiP can be distributed in a preconfigured manner. At the same time, the open design of NanoDiP allows constant addition of reference data and incorporates the pan-cancer functionality of EpiDiP to be run with a laboratory footprint of several square centimetres. The system is intended to run in an offline mode for maximum data security and to prevent changes by (unintended) software updates. In our diagnostic routine we operate our NanoDiP devices behind a hardware-based firewall, allowing only specific IP ranges to communicate with our laboratory information management systems on the local network, reference databases, as well as backup and local monitoring systems. SoCs including breakout boards are at the time of writing priced at about EUR 2000,-- and include all hardware to which the sequencing devices (Mk1B and P2 Solo, ONT, UK) connect with USB ports. Similar (or even lower) pricing applies for all parts to construct a NanoDiP computer from cryptocurrency mining hardware. If needed, NanoDiP also runs in the absence of a sequencer when aimed at data evaluation only. Overall, the proposed hardware platforms are affordable to low-income regions, given that the current WHO CNS Tumour classification [4] incorporates methylation analysis in the "desirable techniques".

User Interface

NanoDiP features a graphical frontend for nanopore sequencer control and data analysis. The user interface (UI) is operated through a local web browser.

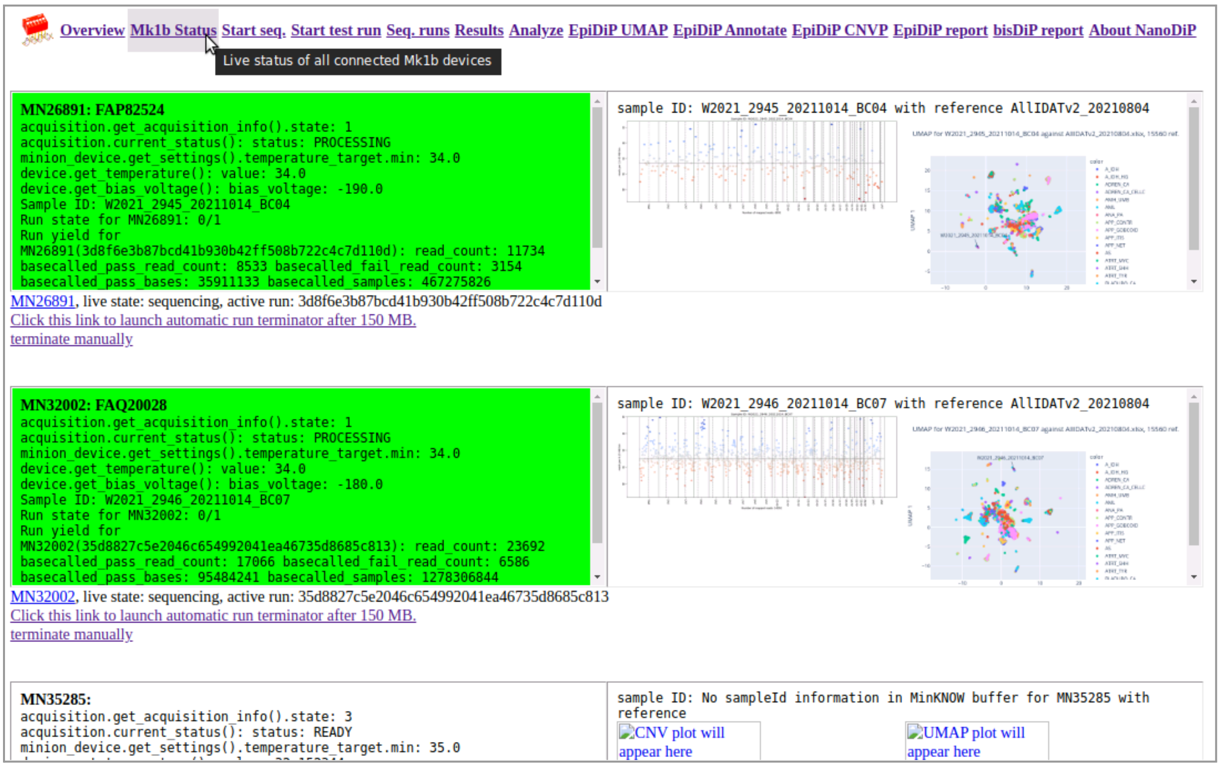

The UI displays in-depth information on attached nanopore sequencers (Mk1B and P2 solo). Idle devices are shown with a white background. Active devices are displayed with a green background with adjacent UMAP and CNV plots calculated based on data acquired. The UI can launch ("Start seq") and stop the sequencing process ("Mk1b Status, "terminate manually" in the field describing each attached sequencer). It can automatically terminate runs upon the acquisition of sufficient data ("Click this link to launch automatic run terminator …"). Already during data acquisition, users may compare the preliminary epigenetic data to reference cohorts of choice and generate copy number plots. Analyses are initiated through "Analyze" and are possible during or after a run on all data present on the NanoDiP device. More connected sequencing devices can be accessed by scrolling down (screenshot above, bottom clipped).

Supplementary Data

MC to SC translation:

The linked sheet contains a methylation class annotation translation table used for the retrospective benchmarking.
https://www.google.com/url?q=https://docs.google.com/spreadsheets/d/1yrJcYzgmFkjX8PXSzpM3z0wHXIvRUdKI/?usp=sharing

Benchmarking MethylSeq vs Microarray UMAP Plot:

Interactive UMAP plot generated with plotly:
20221213_163004_EpiDiP_25000_AllIDATv2_20210804_TWIST_04_AllIDATv2_20210804_TWIST_04.html
https://drive.google.com/file/d/15Ys2PdGp8F-2A-mvm1Gi9CDdyCAlPK5-/view?usp=sharing&e=download
In future versions of EpiDiP/NanoDiP IDATs might be read and processed with SeSaMe [5] or as an alternative to minfi or conumee.

Software Sources

NanoDiP

- https://github.com/neuropathbasel/nanodip (R9, 450K, EPIC V1)
- https://github.com/neuropathbasel/nanodip_dependencies (R9, 450K, EPIC V1)
- https://github.com/neuropathbasel/nanodip_dev (R9, R10 and 450K, EPIC V1,V2)
- https://github.com/neuropathbasel/epidip (legacy website code, 450K, EPIC V1)
- https://github.com/neuropathbasel/methylseqscripts (TWIST MethylSeq Panel)

NanoDiP Demonstration VM

VirtualBox ™ 6.x image, created and tested on Linux hosts
- VM01: R9, 450K, EPIC V1
- VM02: R9, R10, 450K, EPIC V1, V2
- Mirror 1: http or https://www.epidip.org/nanodip_VM/
- Mirror 2: http or https://epidip.usb.ch/nanodip_VM/
- Manual: https://docs.google.com/document/d/1Sd5L6nniXruZS6mSxoy-IJuLwHRP3VSOvy01g2f3ER0/edit?usp=sharing

EPIC V2 Support Source Code References

https://github.com/mwsill/minfi
https://github.com/mwsill/IlluminaHumanMethylationEPICv2manifest
https://github.com/zwdzwd/sesame

MethylSeq Support Source Code References

https://nf-co.re/methylseq
https://github.com/brentp/bwa-meth
https://github.com/dpryan79/methyldackel

Addressing Future Changes of Control and Analysis Software

Users are advised not to update any working version combination of MinKNOW/MinKNOW-API installed locally. This can be enforced by running NanDiP behind a firewall preventing internet access. Current releases of NanoDiP are posted on our GitHub page [www.github.com/neuropathbasel].

Supplementary References

1. Haefliger S, Tzankov A, Frank S, Bihl M, Vallejo A, Stebler J, Hench J (2021) NUT midline carcinomas and their differentials by a single molecular profiling method: a new promising diagnostic strategy illustrated by a case report. Virchows Arch Int J Pathol 478:1007–1012. doi: 10.1007/s00428-020-02869-7
2. Hench J, Vlajnic T, Soysal SD, Obermann EC, Frank S, Muenst S (2022) An Integrated Epigenomic and Genomic View on Phyllodes and Phyllodes-like Breast Tumors. Cancers 14:667. doi: 10.3390/cancers14030667
3. Saleh C, Jaszczuk P, Hund-Georgiadis M, Frank S, Cordier D, Hench IB, Todea A, Wasilewski A, Wilmes S, Grigioni G, Hench J (2020) Differentiation of rare brain tumors through unsupervised machine learning: Clinical significance of in-depth methylation and copy number profiling illustrated through an unusual case of IDH wildtype glioblastoma. Clin Neuropathol. doi: 10.5414/NP301305
4. WHO Classification of Tumours Editorial Board (2021) Central nervous system tumours, 5th edition. International Agency for Research on Cancer, Lyon (France)
5. Zhou W, Triche TJ, Laird PW, Shen H (2018) SeSAMe: reducing artifactual detection of DNA methylation by Infinium BeadChips in genomic deletions. Nucleic Acids Res. doi: 10.1093/nar/gky691
